# Clinical Risk and Overall Survival in Patients with Diabetes Mellitus, Hyperglycemia and Glioblastoma Multiforme. A Review of the Current Literature

**DOI:** 10.3390/ijerph17228501

**Published:** 2020-11-17

**Authors:** Nicola Montemurro, Paolo Perrini, Biagio Rapone

**Affiliations:** 1 Department of Neurosurgery, Azienda Ospedaliera Universitaria Pisana (AOUP), 56126 Pisa, Italy; paolo.perrini@unipi.it; 2 Department of Translational Research and of New Surgical and Medical Technologies, University of Pisa, 56126 Pisa, Italy; 3 Department of Basic Medical Sciences, Neurosciences and Sense Organs, “Aldo Moro” University of Bari, 70121 Bari, Italy; biagiorapone79@gmail.com

**Keywords:** glioblastoma, brain tumors, diabetes, hyperglycemia, type 2 diabetes mellitus, metformin, overall survival, risk factors, ketogenic diet

## Abstract

The relationship between type 2 diabetes mellitus (DM2) and hyperglycemia with cancer patients remains controversial also in the setting of patients with glioblastoma multiforme (GBM), the most common and aggressive form of astrocytoma with a short overall survival (OS) and poor prognosis. A systematic search of two databases was performed for studies published up to 19 August 2020, reporting the OS of patients with DM2 or high blood sugar level and GBM and the clinical risk of diabetic patients for development of GBM. According to PRISMA guidelines, we included a total of 20 papers reporting clinical data of patients with GBM and diabetes and/or hyperglycemia. The aim of this review was to investigate the effect of DM2, hyperglycemia and metformin on OS of patients with GBM. In addition, we evaluated the effect of these factors on the risk of development of GBM. This review supports accumulating evidence that hyperglycemia, rather than DM2, and elevated BMI are independent risk factors for poor outcome and shorter OS in patients with GBM. GBM patients with normal weight compared to obese, and diabetic patients on metformin compared to other therapies, seems to have a longer OS. Further studies are needed to understand better these associations.

## 1. Introduction

Type 2 diabetes mellitus (DM2) is a condition of relative insulin deficiency and beta cell dysfunction associated with obesity and metabolic syndrome. It is the most common chronic illnesses worldwide, affecting 347,000,000 people all over the world and nearly 10% of the population of the USA [1,2,3]. DM2 and hyperglycemia are recognized as independent risk factors in several types of tumor, including cancer of the breast, endometrium, pancreas and liver [4,5,6,7,8]. Some clinical studies reported that DM2 and hyperglycemia lead to excess morbidity and mortality in acute and chronic disease [9,10,11,12,13], but also that they decrease overall survival (OS) of patients with several types of tumors, including breast, colorectal and brain tumors [14,15].

This link remains particularly controversial in patients with glioblastoma multiforme (GBM), the most common and aggressive form of astrocytoma, with a mean OS of 13–15 months in patients underwent surgery [16,17]. OS can be increased till 18.5 months or few months more in patients treated with more than one surgery (second surgery) and standard adjuvant therapy, that is chemotherapy with temozolomide (TMZ) and radiotherapy [18]. Despite improvements in surgical technologies and an increased attention to performed gross total resection (GTR) [19], recurrence occurs almost in the totality of cases, despite adjuvant therapy, intraoperative direct electrical stimulation in awake surgery [20] or new efficient and safe treatments like laser interstitial thermal therapy [21]. It makes sense that it is urgent to find new therapeutic drugs that can increase the survival of these patients.

Genetic mutations and molecular tumor markers became integral part of GBM assessment in modern neuro-oncology [22]. Nowadays, the well-known genetic mutations like O6-methylguanine methyltransferase (MGMT) promoter methylation, which is associated with a significantly higher median survival after therapy with TMZ [23] and other molecular tumor biomarkers, such as epidermal growth factor receptor (EGFR) amplification, aldehyde dehydrogenase 1A3 (ALDH1A3) and isocitrate dehydrogenase (IDH1/IDH2) isoforms are currently foci of research and were also linked to prognosis [22,24]. Limited data exist on the prevalence of DM2 among patients with GBM. Recent works [25,26,27] identified that DM2 is up to 16% of patients with GBM.

Recent evidence suggested that metformin reduces tumor risk in diabetic patients [28] and shows a distinct anti-proliferative effect on glioma cells in vitro and in vivo [29,30]. However, all these associations are constantly updated and diabetes status, glycemic control and use of common antidiabetic drugs in patients with GBM are continuously challenging. In addition, patients with GBM are at particular risk for hyperglycemia, because high-dose glucocorticoids, which are known to increase plasma glucose impairing glucose transport, are routinely used to treat peritumoral edema [31,32].

For this reason, we did a review of the current literature about published papers reporting OS or clinical risk association of diabetic patients or patients with high blood sugar level and GBM. The primary objective of this study was to examine the relationship of OS with (1) DM2 and hyperglycemia, and with (3) metformin in patients with GBM, in order to identify potential therapies for these patients, whereas the secondary objective was to examine if (4) DM2 and hyperglycemia can increase the risk to develop GBM.

## 2. Materials and Methods

### 2.1. Literature Search

A Pubmed and Ovid EMBASE search was performed to identify articles from the period 2000 to present relevant to DM2, hyperglycemia and GBM. PRISMA guidelines (Preferred Reporting Items for Systematic Reviews and Meta-analyses) were followed [33]. The key words “diabetes mellitus”, “diabetes”, “diabetes type 2”, “hyperglycemia”, “glioblastoma” and “glioblastoma multiforme” were used in both “AND” and “OR” combinations. The key words and the detailed search strategy are reported in Table 1.

The inclusion criteria were the following: case series or clinical studies reporting the OS of patients with GBM and clinical data about (1) DM2, (2) hyperglycemia, (3) obesity and/or (4) metformin use. Exclusion criteria were the following: (1) studies published in languages other than English with no available English translations, (2) review articles, (3) case series reporting no data of these clinical risk factors or OS in patients with GBM, (4) studies that not involve human beings, (5) studies with insufficient data about these topics.

### 2.2. Data Collection

From each study, we extracted the following: (1) if DM2 and hyperglycemia are risk factors to develop GBM, (2) if DM2 can affect the OS of patients with GBM, (3) if obesity can affect the OS of patients with GBM, (4) if hyperglycemia can affect the OS of patients with GBM, (5) if the use of metformin can affect the OS of patients with GBM.

### 2.3. Outcomes

The primary objective of this systematic review was to examine the relationship between DM2 and hyperglycemia (risk factors) and GBM. The secondary objectives were to examine the relationship of OS with (1) diabetes, (2) hyperglycemia, (3) BMI and with (4) metformin in patients with GBM.

### 2.4. Quality Scoring

A modified version of the Newcastle–Ottawa Scale [34] was used for the quality assessment of the included studies. Two authors performed the quality assessment independently and the senior author solved discrepancies.

## 3. Results

### 3.1. Literature Review

**Figure 1:**
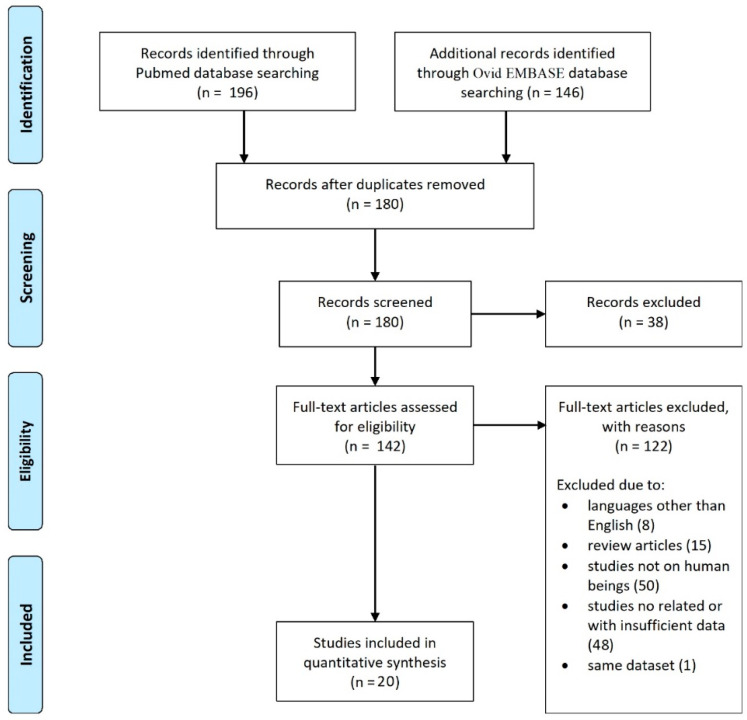
PRISMA flow diagram.

The database search yielded 342 articles. After the removal of duplicates, 180 articles were eligible for screening. A total of 20 articles met the selection criteria [2,25,32,35,36,37,38,39,40,41,42,43,44,45,46,47,48,49,50,51]. Studies included in our systematic review are summarized in Table 2. The search flow diagram is shown in Figure 1.

### 3.2. Quality of Studies

There was complete agreement between the two reviewers for the examined articles. Fourteen studies were retrospective single-center designed, whereas six studies were multicentric investigations. All 20 papers were rated as “high quality” (Table 3).

### 3.3. Risk Factors in the Etiology of GBM

Three papers [2,37,41] out of twenty provided details about DM2 and hyperglycemia and the clinical risk to develop GBM. Barami et al. [37] and Disney-Hogg et al. [41] found a null relationship between these risk factors, whereas Seliger et al. [2] were the only authors who reported a statistically significant decreased risk to develop GBM in patients with DM2 and hyperglycemia. This risk to the develop GBM was lower in both male and female with DM2 and hyperglycemia (OR 0.69). This risk was even lower considering only male patients with DM2 (OR 0.60).

### 3.4. Impact of DM2 and Comorbidities on Overall Survival

Eleven papers [25,35,37,38,39,43,44,45,46,47,51] out of twenty provided clinical information about DM2 impact on OS in patients with GBM, counting for a total of 9795 patients. Among these papers, eight studies [25,35,37,42,43,45,46] reported a null relationship between DM2 and OS in patients with GBM, whereas two papers [37,39] found a decreased OS in patients with DM2 and one paper reported a decreased OS and PFS in patients with DM2 [38]. Four studies [25,35,38,51] reported details about OS in diabetic and non-diabetic patients with GBM, counting for 219 diabetic patients and 1445 non-diabetic patients in total. The weighted mean OS resulted 12.14 months in diabetics compared to 8.69 months in non-diabetic patients.

Twelve studies [32,35,37,38,40,42,43,44,46,49,50,51], counting for a total of 5634 patients, reported data about the effect of hyperglycemia on survival in patients with GBM; 10 papers (83.3%) showed a decreased OS in patients with GBM and hyperglycemia. Chambless et al. [38] reported a decreased OS and PFS in 171 GBM patients with hyperglycemia, whereas Adeberg et al. [35] found just a decreased PFS, but not a null relationship about OS in their 276 GBM patients with hyperglycemia. Table 2 shows that different papers reported different cut-off and in turn it was not possible to obtain overall mean results. However Chambless et al. [38] and McGirt et al. [44] reported a decreased OS in patients with blood glucose values > 180 mg/dl (18.2 and 11 months, respectively) compared to patients with blood glucose values < 180 mg/dl (10.8 and 5 months, respectively), which was statistically significant (*p* < 0.01). Tieu and colleagues [50] reported a statistically significant relationship (*p* = 0.005) between blood glucose values lower or higher than 113 mg/dl and decreased OS (16 vs. 13 months, respectively). Similarly, Stevens et al. [49] reported that blood glucose values > 90 mg/dl decreased OS (7.9 months) compared to patients with blood glucose values < 90 mg/dl (14 months) (*p* < 0.01). Derr et al. [32] showed a progressive decrease in OS as blood glucose values increased, reporting that OS was 14.5 months for blood glucose values lower than 94 mg/dl, 11.6 months (between 94–137 mg/dl) and 9.1 months for blood glucose values higher than 137 mg/dl.

Four papers [36,38,45,47] reported data about the relationship between obesity and OS of patients with GBM. A decreased OS was observed in GBM patients with high Body Mass Index (BMI) in two studies [38,47]. Potharaju and colleagues [45] reported an improved OS in patients with elevated BMI. Three studies [38,44,46] reported details about OS and number of normal weight and obese patients with GBM, counting for 573 patients with normal weight and 305 obese patients. The weighted mean OS resulted 14.46 months in patients with normal weight compared to 12.39 months in obese patients.

A clinical connection between OS and the use of metformin in diabetic patients with GBM was assessed in 4 studies [35,46,48,51], counting for a total of 3003 patients. Three papers [35,48,51] (1272 patients in total) found a better OS in patients with GBM who usually use metformin compared to those patients who do not take it. A single paper [46], counting for 1731, reported a null relationship between metformin and OS in GBM patients. Including the overall patients of papers with data and comparing 38 patients with GBM treated with metformin for DM2 and 125 diabetic patients treated with other therapies except metformin, the weighted mean OS resulted 10.07 and 5.79 months, respectively. Detailed data were reported in Table 2.

## 4. Discussion

### 4.1. The Clinical Risk of DM2 to Developed GBM

The biological mechanism underlying the increased risk of tumor development in patients with diabetes is not well understood [52]. In our review, three papers [2,37,41] provided details about DM2 and hyperglycemia and the clinical risk to develop GBM. Although Barami et al. [37] and Disney-Hogg et al. [41] reported no increased risk of development of glioma in patients with DM2 and hyperglycemia, Seliger and colleagues [2] reported that diabetes was associated with decreased risk of glioma, particularly GBM. This reduction of risk to develop GBM was markedly pronounced among diabetic men, with diabetes of long-term duration or poor glycemic control [2], suggesting that low androgen levels concurrent with diabetes duration and increased glycated hemoglobin (HbA1c) levels as a biologic mechanism [53,54].

A relatively recent study of pre-diagnostic diabetes and cancer at all sites, in 2.3 million Israeli adults [55], found an increased risk of malignant brain tumors among people diagnosed with diabetes. Similarly, Zhao et al. [56] published a meta-analysis reporting the association of DM2 and risk of gliomas of all histologic types and found that DM2 was associated with decreased risk of gliomas in white males. Differently, Tong et al. [57] performed a meta-analysis of 13 studies and observed that diabetics and nondiabetics had similar risk of brain tumors.

### 4.2. The Impact of DM2 and Obesity on Survival in Patients with GBM

In the last years, there is a growing interest in the literature about the impact of DM2 and hyperglycemia on clinical outcome in cancer patients. Diabetes and obesity are associated with an increased requirement for surgical procedures throughout life and furthermore diabetes and obesity themselves represent risk factors for several surgical treatments, as they are associated with increased postoperative morbidity and mortality [58,59,60,61,62]. Adverse outcomes include surgical site infections, impaired wound healing, implant failure and medical complications [59].

Prognostic factors for a longer OS in patients with GBM are young age, good performance status, GTR (tumor resection more than 95%), absence of inflammatory disease or metabolic disease and completion of postoperative radio-chemotherapy [63]. In patients with GBM, the link between DM2 and obesity remains controversial, as previous studies [8,35,64,65] and also our review showed null or negative relationships between GBM and DM2 on OS. Chambless et al. [38] reported that diabetic patients had a decreased median OS (312 vs. 470 days) and a decreased PFS (106 vs. 166 days) compared to non-diabetics, and that an increasing BMI was associated with decreased median PFS. Similarly, Welch & Grommes [51] reported a shorter OS in diabetic patients (10 months) compared with nondiabetic (13.4 months). Several papers [25,35,37,43,44,46,47] did not found any positive or negative relationship (null relationship) between DM2 and GBM in term of survival. No papers reported that having diabetes can increase OS. Siegel et al. [47] found a null relationship between DM and OS, whereas OS decreases among patients underweight (median OS: 12.0 months) or obese (median OS: 13.6 months) when compared to patients of normal weight (median OS: 17.5 months). However, this appears in contrast with the findings of Potharaju et al. [45]. In their series of 392 patients with GBM [45], the median OS was 13.5 months in normal subjects, 15.4 months in overweight subjects and 15.1 months in obese subjects, concluding that patients with GBM and elevated BMIs had a significantly better OS. Even if not well understood, this phenomenon, also known as “the obesity paradox”, seems to be more and more common in the last years in patients with tumors, as various studies suggested that obese patients with lung cancer, renal cell carcinoma and melanoma have a better outcomes compared to non-obese patients [66]. From this review, the weighted mean OS results 14.46 months in patients with normal weight compared to 12.39 months in obese patients.

### 4.3. The Impact of Hyperglycemia on Survival in Patients with GBM

The relationship between hyperglycemia with cancer patients has been evaluated in several previous studies, reporting a positive, null, or inverse relation between DM2 and its associated factors [4]. Although some studies [38,51] have shown a possible negative correlation between DM2 and GBM on OS, 7 out of 10 (70%) showed a null relationship. On the other hand, 10 out of 12 papers (83.3%) reported a decreased OS in patients with GBM with hyperglycemia, supporting that high blood sugar levels can affect the OS and PFS in patients with GBM. As literature reported that hyperglycemia is an independent risk factor for decreased survival in different diseases and tumors [37], this review confirmed that hyperglycemia decreased OS also in patients with GBM.

This shorter OS in patients with GBM and hyperglycemia is found also in patients with complete tumor resection and adjuvant treatment [43]. Mayer et al. [43] reported that the occurrence of one or more deregulated blood glucose values more than 10 mM is associated with a reduction in median OS from 16.7 to 8.8 months in patients with GBM. Similarly McGirt et al. [44] concluded that persistent high value of blood glucose was associated with decreased OS in patients undergoing surgical resection and that it was independent of the degree of disability, diabetes, prolonged dexamethasone use and subsequent treatment modalities.

Derr et al. [32], dividing patients into quartiles (glucose levels ranged between 65 and 459 mg/dL), showed that median OS decreased as blood glucose values increased and that this association between higher blood glucose levels and shorter OS persisted after adjustment for daily glucocorticoid dose, age and Karnofsky performance score. Stevens and colleagues [49] reported in their study on 242 GBM patients that a higher preoperative glucose level was associated with decreased OS in patients diagnosed with GBM, regardless of whether or not patients underwent GTR and that strict glucose control may contribute to improve the outcome of these patients. Tieu et al. [50] demonstrated that glycemia remains an independent predictor for survival in GBM patients treated with surgical resection and subsequently with RT and TMZ.

Hagan et al. [42] showed in a multivariate analysis a statistically significant decrease in OS for plasma glucose concentrations > 112 mg/dL and > 180 mg/dL (*p* = 0.01) in the preoperative period in patients with GBM, although no statistically significant association between hyperglycemia and PFS was found. Decker et al. [40] analyzed the mechanisms underlying the relationship between hyperglycemia and worse OS and found that median blood glucose values greater than 167 mg/dL were associated with increased serious post-operative complications and values greater than 163 mg/dL were associated with increased 30-day readmissions in hospital. Post-operative hyperglycemia in patients with GBM put patients in a vulnerable condition to develop post-operative complications and readmissions that potentially delaying further treatment of their disease.

Welch & Grommes [51] reported that patients with a good glycemic control (median glucose ≤ 173 mg/dl) had a better median OS of 11 months, compared to patients with glucose value from 174 to 247 mg/dl (OS of 9 months) and with median glucose was ≥ 247 mg/dl (OS of 8 months). However, larger patient cohorts are needed for further evaluation of the role of hyperglycemia and BMI in patients with GBM. The use of telemedicine is increasing worldwide for diabetes management and patients with brain tumor [67,68,69] and this can potentially improve blood glucose control and clinical outcomes of these patients.

Although two papers [44,46] reported a null relationship, we did not find any papers showing an improved OS in patients with GBM and high blood sugar level, supporting the hypothesis that high blood glucose levels can actually worsen prognosis of these patients. For this reason, an increased glucose control has to be taken into account in patients with GBM as it may contribute to improved OS and outcomes in these patients.

The effect of hyperglycemia on GBM cell is not well known, although it seems that several potential mechanisms are involved. Hyperglycemia triggers intracellular pathways, which promote tumor progression, such as increased leptin levels and pro-cell survival AKT/mTOR, enhancement of WNT/βcatenin signaling, induction of epithelial mesenchymal transition and upregulation of inflammatory cytokine levels in circulation [70,71,72]. Many pathways long known to be associated with tumor cell growth, escape from apoptosis, aggressive blood vessel formation (angiogenesis) and therapy resistance have recently been linked to cellular metabolism [73], as for example p53 which is tumor suppressor encoded by the human gene TP53. p53, frequently mutated in GBM patients, promotes a variety of cellular responses to hypoxia, DNA damage and oncogene activation [25], but also regulates glycolysis and assist in maintaining mitochondrial integrity [74]. It seems that an over-activation of the stress responsive PI3K/AKT signaling pathway is closely linked to metabolism and that, under low glucose conditions, it results in rapid tumor cell death [75]. Furthermore, hyperglycemia enhanced the functional expression of a G protein coupled formylpeptide receptor 2 (FPR2). A variant of FPR2, FPR1 is expressed on human GBM cells in xenograft models and in human specimens and is associated with poorer patient survival [70,76,77]. Zhou et al. [77] reported that human GMB cells in condition of hyperglycemia express increased levels of FPR1, appearing to mediate motility, growth, and angiogenesis of human GBM, as well as the receptor for EGF (EGFR), in association with more rapid tumor progression in diabetic animals. FPR may represent a molecular target for the development of novel glioma therapeutics [77].

### 4.4. The Impact of Metformin and Other Oral Antidiabetic Drugs on GBM Patients

Tumor cells reliance on glucose suggests that treatments affecting cellular metabolism may be an effective method to improve current therapies [78]. Common classes of oral antidiabetic drugs are biguanides (metformin), sulfonylureas (glimepiride), meglitinides (repaglinide) and thiazolidinediones (pioglitazone) [79]. Several observational studies [80,81] in the treatment of cancer patients showed the role of metformin and thiazolidinediones (PPAR-g agonists) in improving the survival of patients with tumors. On the other hand, different studies [50,82] reported a poorer OS among patients with tumors treated with sulfonylureas.

Metformin, best known as a growth inhibitor, induces glucose stabilization, enhances insulin sensitivity and reduces the circulating insulin levels without associated hypoglycemia [48]. In recent years, interest about how DM2 and antidiabetic drugs affects survival of patients with GBM [51] is growing. In patients with GBM seems that the use of metformin is associated with a better OS, while sulfonylureas may be associated with a poor outcome. Welch and Grommes [51] reported that GBM patients that received metformin had an improved median OS compared with patients receiving other antidiabetic medications (insulin monotherapy, thiazolidinedione and sulfonylureas). Welch & Grommes [51] showed a clear survival benefit with the use of metformin. In fact, patients treated with metformin had a median OS of 10 months whereas patients treated other monotherapies had an OS of 8 months. Patients treated with sulfonylureas had worse outcomes with a median OS of only 6 months [51]. This statistically significant difference remains considering the subpopulation of only diabetic GBM patients who received surgery, radiation and chemotherapy [50]. In this subpopulation, OS was 14 months in patients treated with metformin compared to patients treated with other antidiabetic drugs (8 months) [51]. Our review found that patients with GBM treated with metformin for DM2 had a longer OS (10.1 months) compared to diabetic patients treated with other therapies except metformin (5.79 months).

Several studies reported that metformin could represent a potential enhancer of the cytotoxic effects of TMZ and/or radiotherapy. Soritau and colleagues [48] showed a good clinical link in term of OS in patients treated with TMZ plus metformin compared to those patients treated with TMZ alone, suggesting that metformin enhance the effect of TMZ.

Biological and functional activity of metformin against oncogenesis is not well known, but is thought to induce AMP-activated protein kinase activity and inactivates the mammalian target of rapamycin (mTOR), S6 kinase and mRNA translation [48]. Sesen and colleagues [83] demonstrated that metformin decreases mitochondrial-dependent ATP production and oxygen consumption and increases lactate and glycolytic ATP production, showing that metformin induces decreased proliferation, cell cycle arrest, autophagy, apoptosis and cell death in vitro and in human GBM cells. Metformin may inhibit glioma cell proliferation, migration and invasion, and promote its apoptosis. These effects may be associated with the AMPK/mTOR signaling pathway and oxidative stress [83].

Xiao et al. [84] demonstrated that repaglinide significantly inhibited the proliferation and migration of human GBM cells in vitro and that prominently prolonged the median survival time of mice bearing orthotopic glioma in vivo. In vivo repaglinide prominently prolonged the median survival time of mice bearing orthotopic glioma, reducing Bcl-2, Beclin-1 and PD-L1 expression in glioma tissues and indicating that repaglinide may exert its anti-cancer effects via apoptotic, autophagic and immune checkpoint signaling [84]. Additional studies on the mechanisms involved in the antineoplastic effects of oral antidiabetic drugs are needed.

### 4.5. The Impact of Steroid on Survival in Patients with GBM

Steroid-induced hyperglycemia is a relevant topic since as increasing number of clinical studies unequivocally demonstrated a worse prognosis associated with high blood glucose levels in patients with glioma [32,35,43,50,51,85,86]. Steroids are used perioperatively to treat cerebral peritumoral edema and to reduce intracranial pressure and are administered during subsequent treatments, to reduce the side effects [87].

However, the effects of corticosteroids in vivo and in vitro remained controversial [88]. Steroids may decrease the effectiveness of treatment and reduce OS in patients with GBM, as elevated blood glucose levels would contribute to radioresistance of GBM cells through several mechanisms [88,89]. On the other hand, it seems that dexamethasone-induced anti-proliferative effects may confer protection from radiotherapy- and chemotherapy-induced genotoxic stress [88]. For this reason, Pitter et al. [89] suggested the importance of identifying alternative agents such as vascular endothelial growth factor (VEGF) antagonists to control peritumoral edema. Adeberg et al. [35] and Welch & Grommes [51] reported a strong relationship between survival and steroid dependency and a poor outcome in patients with steroid dependency (*p* < 0.001). Welch and Grommes [51] showed that patients who remained steroid dependent (85% of patients) had a shorter median OS (9 months) compared to patients who subsequently stopped steroids (17 months). However, Adeberg et al. [35] reported that a limitation of their study was that the evaluation of corticosteroid therapy was non-standardized and mainly collected from discharge reports and patient charts; for this reason, patients with short-term corticosteroid therapy might remain undetected and influence the analysis. The duration of steroid therapy and the daily dose can vary significantly between patients and between institutions, as some institutions are more likely to reduce steroids sooner than others. However in both papers [35,51], it is difficult to understand if patients who remain steroid dependent have a decreased OS due to the side effects of steroid use or due to the fact those patients with more extensive GBM or with GBM located in eloquent areas or patients who did not underwent GTR (all well know conditions that reduced OS) require steroids therapy till death. Welch & Grommes [51] showed that steroid dependency often correlates with tumor burden and that the 8-month survival benefit they found among patients weaned from dexamethasone was, in part, a reflection of less aggressive disease. Some studies [44,88], reported that steroid-induced hyperglycemia has a negative prognostic influence irrespective of tumor size. Pitter et al. [88] showed that the use of corticosteroids early in the course of disease, during radiotherapy without or with chemotherapy, is an independent predictor of poor outcome in three independent patient cohorts, where age, gender, duration of symptoms or TMZ use between patients that did or did not receive steroids at the start of therapy were similar. The effect of glucocorticoid-related hyperglycemia on OS deserves additional study in patients with GBM.

### 4.6. Ketogenic Diet

Recently, the ketogenic diet has been proposed as an adjunct treatment for a range of medical conditions including weight loss, diabetes, neurodegenerative diseases and tumors. Because malignant CNS tumors are highly dependent on glucose, the use of a ketogenic diet as an adjunct therapy is currently being explored [90]. Ketogenic diet limits carbohydrate intake, lower glucose levels and reduces insulin and insulin-like growth factor (IGF) levels [25,78]. In addition, the increased levels of ketone from baseline results in a reduction in BMI and in a reduction in vasogenic peritumoral edema [90]. Ketogenic therapy for CNS tumors, including glioma, extends far beyond the originally proposed mechanism of reducing glucose availability and may work through multiple mechanisms such as reducing inflammation and the oxidative stress [91,92]. Some trial was published about this topic [93,94], but further studies are needed to assess the real benefit of ketogenic diet on survival of patients with GBM.

### 4.7. Limitations of the Study

Our review has several limitations. The series reported are mostly retrospective and single-institution experiences. Second, our review might be underpowered because this topic is seldom reported in the literature and subjected to continuous updating. Third, although DM2 seems to affect survival only in 2 out of 10 studies, it is extremely difficult to obtain comprehensive data and we are unable to know if patients with DM2 had their blood sugar under control or not and whether these patients had high BMIs. Lastly, studies varied in the use of systemic therapies, other comorbidities, extent of surgery and size and site of tumors. For this reason, larger studies are required to analyze the relationship between DM2 and hyperglycemia in GBM patients.

## 5. Conclusions

Hyperglycemia, rather than DM2, and elevated BMI are independent risk factors for poor outcome and shorter OS in patients with GBM. GBM patients with normal weight compared to obese, and diabetic patients on metformin compared to other therapies, seems to have a longer OS. The mechanism of this interaction needs to be explored further to understand better these associations or interconnections. An increased glucose control has to be warranted in patients with GBM as it may contribute to improve the OS and the outcome in these patients.

## Figures and Tables

**Table 1:** Search syntax.

PubMed Search Accessed on 19 August 2020 (196 Articles)	Embase Search Accessed on 19 August 2020 (146 Articles)
(diabetes mellitus OR diabetes OR diabetes type 2 OR hyperglycemia) AND (glioblastoma OR glioblastoma multiforme)	(‘diabetes mellitus’ OR ‘diabetes’ OR ‘diabetes type 2’ OR ‘hyperglycemia’) AND (‘glioblastoma’ OR ‘glioblastoma multiforme’)

**Table 2:** Summary of studies included in the review.

Studies	Year	N° of Patients	Topics and Findings					
			DM2 and Risk of GBM	Hyperglycemia and Risk of GBM	DM2 and Survival of Patients with GBM (Median OS in Months; 95% CI)	Hyperglycemia and Survival of Patients with GBM (Median OS in Months; 95% CI)	Obesity and Survival of Patients with GBM (Median OS in Months; 95% CI)	Metformin Use and Survival of Patients with GBM (Median OS in Months; 95% CI)
McGirt et al. [44]	2008	297	-	-	**Null relationship**	**Decreased OS** <180 mg/dl (11), >180 mg/dl (5) *p* = 0.001	-	-
Derr et al. [32]	2009	191	-	-	-	**Decreased OS** <94 mg/dl (14.5), 94–137 mg/dl (11.6), >137 mg/dl (9.1) *p* = 0.041 for trend	-	-
Grommes et al. [25]	2010	229	-	-	**Null relationship** diabetics (6; 3–8), non-diabetics (8; 7–11) *p* = 0.18	-	-	-
Jones et al. [37]	2010	1259	-	-	-	-	**Null relationship**	-
Soritau et al. [48]	2011	8	-	-	-	-	-	**Improved OS in association with TMZ** *p* < 0.05
Chambless et al. [38]	2012	171	-	-	**Decreased OS** OS: diabetics (10.4), non-diabetics (15.7) *p* = 0.003 **Decreased PFS** PFS: diabetics (3.5), non-diabetics (5.3) *p* = 0.04	**Decreased OS** <180 mg/dl (18.2), >180 mg/dl (10.8) *p* = 0.01	**Decreased PFS** **normal (6.5), overweight (5.5), obese (4.8)** ***p* = 0.003**	-
Stevens et al. [49]	2012	242	-	-	**-**	**Decreased OS** **<90 mg/dl (14.0),** **>90 mg/dl (7.9)** ***p* < 0.001**	**-**	-
Siegel et al. [47]	2013	852	-	-	**Null relationship**	**-**	**Decreased OS** **normal (17.5),** **obese (13.6)** ***p* = 0.004**	-
Welch & Grommes [51]	2013	988	-	-	**Decreased OS** diabetics (10; 8–12), non-diabetics (13.4; 12–14) *p* < 0.001	**Null relationship** 98.5–173 mg/dl (11; 9–17), 174–247 mg/dl (9; 7–12), >247 mg/dl (8; 2–26) *p* = 0.36	-	**Improved OS** metformin use (10), other monotherapies (6) *p* = 0.02
Mayer et al. [43]	2014	106	-	-	**Null relationship**	**Decreased OS** <180 mg/dl (16.7) >180 mg/dl (8.8), *p* = 0.001	-	-
Adeberg et al. [35]	2015	276	-	-	**Null relationship OS** ***p* = 0.06** **Null relationship PFS** diabetics (6.7), non-diabetics (8.5) ***p* = 0.241**	**Decreased OS** ***p* < 0.001** **Null relationship PFS** ***p* = 0.051**	-	**Improved PFS** metformin use (10.13), no metformin use (4.67) *p* = 0.043 **Null relationship OS** *p* = 0.326
Tieu et al. [50]	2015	393	-	-	-	**Decreased OS** **<113 mg/dl (16),** **>113 mg/dl (13)** ***p* = 0.005**	-	-
Seliger et al. [2]	2016	852	Decreased risk of GBM OR = 0.69 Decreased risk of GBM in males OR = 0.60	Decreased risk of GBM in males OR = 0.20 (HbA1c ≥8 vs. <6.5%)	-	-	-	-
Barami et al. [37]	2017	969	**Null relationship** *p* = 0.9	**Null relationship** *p* = 0.09	**Null relationship** *p* < 0.1	**Decreased OS** **HbA1c < or > 6.9%** *p* < 0.01	-	-
Chen et al. [39]	2017	3784			Decreased OS HR 1.175 (1.075–1.284) *p* < 0.05			
Hagan et al. [42]	2017	162	-	-		Decreased OS > or <112 mg/dL *p* = 0.01 Null relationship PFS *p* = 0.33	-	-
Disney-Hogg et al. [41]	2018	na	**Null relationship** *p* = 0.9	**Null relationship** *p* = 0.9	-	-	-	-
Potharaju et al. [45]	2018	392	-	-	**Null relationship** *p* = 0.3	-	**Improved OS** normal (13.5), overweight (15.4), *p* < 0.001 normal (13.5), obese (15.1) *p* = 0.035	-
Decker et al. [40]	2019	108	-	-	-	Decreased OS blood glucose value < or >167 mg/dL *p* = 0.0087	-	-
Seliger et al. [46]	2020	1731	-	-	Null relationship OS *p* = 0.936 **Null relationship PFS** *p* = 0.585	Null relationship Non-fasting blood glucose ≤ or >200 mg/dl *p* = 0.979	-	Null relationship OS *p* = 0.364 Null relationship PFS *p* = 0.072

CI, confidence interval; DM2, Type 2 Diabetes Mellitus; GBM, glioblastoma; -, not available; HbA1c, Hemoglobin A1c; HR, hazard ratio; OR, odd ratio; OS, overall survival; PFS, progression-free survival; TMZ, temozolomide.

**Table 3:** Quality measure of included studies by the Newcastle–Ottawa quality assessment scale.

Study	Selection				Comparability		Outcome			TOT
	(1)	(2)	(3)	(4)	(a)	(b)	(1)	(2)	(3)	
McGirt et al. [44]	*	*	*		*	*	*	*		7
Derr et al. [32]	*	*	*		*	*	*	*		7
Grommes et al. [25]	*	*	*		*	*	*	*		7
Jones et al. [36]	*	*	*		*	*	*	*		7
Soritau et al. [48]	*	*	*		*	*	*	*		7
Chambless et al. [38]	*	*	*		*	*	*	*		7
Stevens et al. [49]	*	*	*		*	*	*	*		7
Siegel et al. [47]	*	*	*		*	*	*	*		7
Welch & Grommes [51]	*	*	*		*	*	*	*		7
Mayer et al. [43]	*	*	*		*	*	*	*		7
Adeberg et al. [35]	*	*	*		*	*	*	*		7
Tieu et al. [50]	*	*	*		*	*	*	*		7
Seliger et al. [2]	*	*	*		*	*	*	*		7
Barami et al. [37]	*	*	*		*	*	*	*		7
Chen et al. [39]	*	*	*		*	*	*	*		7
Hagan et al. [42]	*	*	*		*	*	*	*		7
Disney-Hogg et al. [41]	*	*	*		*	*	*	*		7
Potharaju et al. [45]	*	*	*		*	*	*	*		7
Decker et al. [40]	*	*	*		*	*	*	*		7
Seliger et al. [46]	*	*	*		*	*	*	*		7

* present.

## References

[1] Danaei G., Finucane M.M., Lu Y., Singh G.M., Cowan M.J., Paciorek C.J., Lin J.K., Farzadfar F., Khang Y., Stevens G.A. (2011). National, regional, and global trends in fasting plasma glucose and diabetes prevalence since 1980: Systematic analysis of health examination surveys and epidemiological studies with 370 country-years and 2·7 million participants. Lancet.

[2] Seliger C., Ricci C., Meier C.R., Bodmer M., Jick S.S., Bogdahn U., Hau P., Leitzmann M.F. (2016). Diabetes, use of antidiabetic drugs, and the risk of glioma. Neuro Oncol..

[3] Kaul K., Tarr J.M., Ahmad S.I., Kohner E.M., Chibber R. (2012). Introduction to diabetes mellitus. Adv. Exp. Med. Biol..

[4] Heidemann C., Boeing H., Pischon T., Nöthlings U., Joost H.G., Schulze M.B. (2009). Association of a diabetes risk score with risk of myocardial infarction, stroke, specific types of cancer, and mortality: A prospective study in the European Prospective Investigation into Cancer and Nutrition (EPIC)-Potsdam cohort. Eur. J. Epidemiol..

[5] Saltzman B.S., Doherty J.A., Hill D.A., Beresford S.A., Voigt L.F., Chen C., Weiss C.N. (2008). Diabetes and endometrial cancer: An evaluation of the modifying effects of other known risk factors. Am. J. Epidemiol..

[6] Ben Q., Cai Q., Li Z., Yuan Y., Ning X., Deng S., Wang K. (2011). The relationship between new-onset diabetes mellitus and pancreatic cancer risk: A case-control study. Eur. J. Cancer..

[7] Wang C.S., Yao W.J., Chang T.T., Wang S.T., Chou P. (2009). The impact of type 2 diabetes on the development of hepatocellular carcinoma in different viral hepatitis statuses. Cancer Epidemiol. Biomark. Prev..

[8] Campbell P.T., Newton C.C., Patel A.V., Jacobs E.J., Gapstur S.M. (2012). Diabetes and cause-specific mortality in a prospective cohort of one million U.S. adults. Diabetes Care..

[9] Van den Berghe G., Wouters P., Weekers F., Verwaest C., Bruyninckx F., Schetz M., Vlasselaers D., Ferdinande P., Lauwers P., Bouillon R. (2001). Intensive insulin therapy in the critically ill patients. N. Engl. J. Med..

[10] Rapone B., Corsalini M., Converti I., Loverro M.T., Gnoni A., Trerotoli P., Ferrara E. (2020). Does Periodontal Inflammation Affect Type 1 Diabetes in Childhood and Adolescence? A Meta-Analysis. Front. Endocrinol..

[11] Corsalini M., Di Venere D., Rapone B., Stefanachi G., Laforgia A., Pettini F. (2017). Evidence of signs and symptoms of Craniomandibular Disorders in Fibromyalgia patients. Open Dent. J..

[12] Perrini P., Montemurro N., Caniglia M., Lazzarotti G., Benedetto N. (2015). Wrapping of intracranial aneurysms: Single-center series and systematic review of the literature. Br. J. Neurosurg..

[13] Di Venere D., Corsalini M., Nardi G.M., Laforgia A., Grassi F.R., Rapone B., Pettini F. (2017). Obstructive site localization in patients with Obstructive Sleep Apnea Syndrome: A comparison between otolaryngologic data and cephalometric values. Oral Implantol..

[14] Yancik R., Wesley M.N., Ries L.A., Havlik R.J., Edwards B.K., Yates J.W. (2001). Effect of age and comorbidity in postmenopausal breast cancer patients aged 55 years and older. JAMA.

[15] Polednak A.P. (2006). Comorbid diabetes mellitus and risk of death after diagnosis of colorectal cancer: A population-based study. Cancer Detect. Prev..

[16] Müller D.M.J., Robe P.A.J.T., Eijgelaar R.S., Witte M.G., Visser M., de Munck J.C., Broekman M.L.D., Seute T., Hendrikse J., Noske D.P. (2019). Comparing Glioblastoma Surgery Decisions Between Teams Using Brain Maps of Tumor Locations, Biopsies, and Resections. JCO Clin. Cancer Inform..

[17] Bruhn H., Strandéus M., Milos P., Hallbeck M., Vrethem M., Lind J. (2018). Improved survival of Swedish glioblastoma patients treated according to Stupp. Acta Neurol. Scand..

[18] Montemurro N., Perrini P., Blanco M.O., Vannozzi R. (2016). Second surgery for recurrent glioblastoma: A concise overview of the current literature. Clin. Neurol. Neurosurg..

[19] Perrini P., Gambacciani C., Weiss A., Pasqualetti P., Delishaj D., Paiar F., Morganti R., Vannozzi R., Lutzemberger L. (2017). Survival outcomes following repeat surgery for recurrent glioblastoma: A single-center retrospective analysis. J. Neurooncol..

[20] Montemurro N., Herbet G., Duffau H. (2016). Right Cortical and Axonal Structures Eliciting Ocular Deviation during Electrical Stimulation Mapping in Awake Patients. Brain Topogr..

[21] Montemurro N., Anania Y., Cagnazzo F., Perrini P. (2020). Survival outcomes in patients with recurrent glioblastoma treated with Laser Interstitial Thermal Therapy (LITT): A systematic review. Clin. Neurol. Neurosurg..

[22] Montemurro N. (2020). Glioblastoma Multiforme and Genetic Mutations: The Issue Is Not Over Yet. An Overview of the Current Literature. J. Neurol. Surg. A Cent. Eur. Neurosurg..

[23] Bobola M.S., Alnoor M., Chen J.Y., Kolstoe D.D., Silbergeld D.L., Rostomily R.C., Blank A., Chamberlain M.C., Silber J.R. (2015). O6-methylguanine-DNA methyltransferase activity is associated with response to alkylating agent therapy and with MGMT promoter methylation in glioblastoma and anaplastic glioma. BBA Clin..

[24] Carr M.T., Hochheimer C.J., Rock A.K., Dincer A., Ravindra L., Zhang F.L., Opalak C.F., Poulos N., Sima A.P., Broaddus W.C. (2019). Comorbid Medical Conditions as Predictors of Overall Survival in Glioblastoma Patients. Sci. Rep..

[25] Grommes C., Conway D.S., Alshekhlee A., Barnholtz-Sloan J.S. (2010). Inverse association of PPARγ agonists use and high grade glioma development. J. Neurooncol..

[26] Schwartzbaum J., Jonsson F., Ahlbom A., Preston-Martin S., Malmer B., Lönn S., Söderberg K., Feychting M. (2005). Prior hospitalization for epilepsy, diabetes, and stroke and subsequent glioma and meningioma risk. Cancer Epidemiol. Biomark. Prev..

[27] Purow B. (2016). For glioma, a sweet side to diabetes. Neuro Oncol..

[28] Evans J.M., Donnelly L.A., Emslie-Smith A.M., Alessi D.R., Morris A.D. (2005). Metformin and reduced risk of cancer in diabetic patients. BMJ.

[29] Sato A., Sunayama J., Okada M., Watanabe E., Seino S., Shibuya K., Suzuki K., Narita Y., Shibui S., Kayama T. (2013). Glioma-initiating cell elimination by metformin activation of FOXO_3_ via AMPK. Stem Cells Transl. Med..

[30] Wurth R., Pattarozzi A., Gatti M., Bajetto A., Corsaro A., Parodi A., Sirito R., Massollo M., Marini C., Zona G. (2012). Metformin selectively affects human glioblastoma tumor-initiating cell viability: A role for metformin-induced inhibition of Akt. Cell Cycle.

[31] Donihi A.C., Raval D., Saul M., Korytkowski M.T., DeVita M.A. (2006). Prevalence and predictors of corticosteroid-related hyperglycemia in hospitalized patients. Endocr. Pr..

[32] Derr R.L., Ye X., Islas M.U., Desideri S., Saudek C.D., Grossman S.A. (2009). Association between hyperglycemia and survival in patients with newly diagnosed glioblastoma. J. Clin. Oncol..

[33] Moher D., Shamseer L., Clarke M., Ghersi D., Liberati A., Petticrew M., Shekelle P., Stewart L.A., PRISMA-P Group (2015). Preferred reporting items for systematic review and meta-analysis protocols (PRISMA-P) 2015 statement. Syst. Rev..

[34] Wells G.A., Shea B., O’Connell D., Peterson J., Welch V., Losos M., Tugwell P. (2013). The NewcastleOttawa Scale (NOS) for assessing the quality of nonrandomized studies in meta-analyses. Ott. Hosp. Res. Inst..

[35] Adeberg S., Bernhardt D., Ben Harrabi S., Bostel T., Mohr A., Koelsche C., Diehl C., Rieken S., Debus J. (2015). Metformin influences progression in diabetic glioblastoma patients. Strahlenther. Onkol..

[36] Jones L.W., Ali-Osman F., Lipp E., Marcello J.E., McCarthy B., McCoy L., Rice T., Wrensch M., Il’yasova D. (2010). Association between body mass index and mortality in patients with glioblastoma mutliforme. Cancer Causes Control..

[37] Barami K., Lyon L., Conell C. (2017). Type 2 Diabetes Mellitus and Glioblastoma Multiforme-Assessing Risk and Survival: Results of a Large Retrospective Study and Systematic Review of the Literature. World Neurosurg..

[38] Chambless L.B., Parker S.L., Hassam-Malani L., McGirt M.J., Thompson R.C. (2012). Type 2 diabetes mellitusand obesity are independent risk factors for poor outcome in patients with high-grade glioma. J. Neurooncol..

[39] Chen Y.R., Ugiliweneza B., Burton E., Woo S.Y., Boakye M., Skirboll S. (2017). The effect of postoperative infection on survival in patients with glioblastoma. J. Neurosurg..

[40] Decker M., Sacks P., Abbatematteo J., De Leo E., Brennan M., Rahman M. (2019). The effects of hyperglycemia on outcomes in surgical high-grade glioma patients. Clin. Neurol. Neurosurg..

[41] Disney-Hogg L., Sud A., Law P.J., Cornish A.J., Kinnersley B., Ostrom Q.T., Labreche K., Eckel-Passow J.E., Armstrong G.N., Claus E.B. (2018). Influence of obesity-related risk factors in the aetiology of glioma. Br. J. Cancer..

[42] Hagan K., Bhavsar S., Arunkumar R., Grasu R., Dang A., Carlson R., Cowles C., Arnold B., Potylchansky Y., Rahlfs T.F. (2017). Association Between Perioperative Hyperglycemia and Survival in Patients With Glioblastoma. J. Neurosurg. Anesthesiol..

[43] Mayer A., Vaupel P., Struss H.G., Giese A., Stockinger M., Schmidberger H. (2014). Strong adverse prognostic impact of hyperglycemic episodes during adjuvant chemoradiotherapy of glioblastoma multiforme. Strahlenther. Onkol..

[44] McGirt M.J., Chaichana K.L., Gathinji M., Attenello F., Than K., Ruiz A.J., Olivi A., Quiñones-Hinojosa A. (2008). Persistent outpatient hyperglycemia is independently associated with decreased survival after primary resection of malignant brain astrocytomas. Neurosurgery.

[45] Potharaju M., Mangaleswaran B., Mathavan A., John R., Thamburaj V., Ghosh S., Ganesh S., Kalvakonda C., Loganathan M., Bapu S. (2018). Body Mass Index as a Prognostic Marker in Glioblastoma Multiforme: A Clinical Outcome. Int. J. Radiat. Oncol. Biol. Phys..

[46] Seliger C., Genbrugge E., Gorlia T., Chinot O., Stupp R., Nabors B., Weller M., Hau P., EORTC Brain Tumor Group (2020). Use of metformin and outcome of patients with newly diagnosed glioblastoma: Pooled analysis. Int. J. Cancer.

[47] Siegel E.M., Nabors L.B., Thompson R.C., Olson J.J., Browning J.E., Madden M.H., Han G., Egan K.M. (2013). Prediagnostic body weight and survival in high grade glioma. J Neurooncol..

[48] Soritau O., Tomuleasa C., Aldea M., Petrushev B., Susman S., Gheban D., Ioani H., Cosis A., Brie I., Irimie A. (2011). Metformin plus temozolomide-based chemotherapy as adjuvant treatment for WHO grade III and IV malignant gliomas. J. Buon..

[49] Stevens G., Ahluwalia M. (2012). Elevated Preoperative Glucose Levels and Survival in Elderly Newly Diagnosed Glioblastoma Patients (P07.111). Neurology.

[50] Tieu M.T., Lovblom L.E., McNamara M.G., Mason W., Laperriere N., Millar B.A., Ménard C., Kiehl T., Perkins B.A., Chung C. (2015). Impact of glycemia on survival of glioblastoma patients treated with radiation and temozolomide. J. Neurooncol..

[51] Welch M.R., Grommes C. (2013). Retrospective analysis of the effects of steroid therapy and antidiabetic medication on survival in diabetic glioblastoma patients. CNS Oncol..

[52] Giovannucci E., Harlan D.M., Archer M.C., Bergenstal R.M., Gapstur S.M., Habel L.A., Pollak M., Regensteiner J.G., Yee D. (2010). Diabetes and cancer: A consensus report. Diabetes Care..

[53] Rapone B., Ferrara E., Corsalini M., Converti I., Grassi F.R., Santacroce L., Topi S., Gnoni A., Scacco S., Scarano A. (2020). The Effect of Gaseous Ozone Therapy in Conjunction with Periodontal Treatment on Glycated Hemoglobin Level in Subjects with Type 2 Diabetes Mellitus: An Unmasked Randomized Controlled Trial. Int. J. Environ. Res. Public Health.

[54] Di Venere D., Nardi G.M., Lacarbonara V., Laforgia A., Stefanachi G., Corsalini M., Grassi F.R., Rapone B., Pettini F. (2017). Early mandibular canine-lateral incisor transposition: Case report. Oral Implantol..

[55] Dankner R., Boffetta P., Balicer R.D., Boker L.K., Sadeh M., Berlin A., Olmer L., Goldfracht M., Freedman L.S. (2016). Time-Dependent Risk of Cancer after a Diabetes Diagnosis in a Cohort of 2.3 Million Adults. Am. J. Epidemiol..

[56] Zhao L., Zheng Z., Huang P. (2016). Diabetes mellitus and the risk of glioma: A meta-analysis. Oncotarget.

[57] Tong J.J., Tao H., Hui O.T., Jian C. (2012). Diabetes mellitus and risk of brain tumors: A meta-analysis. Exp. Med..

[58] Montemurro N., Murrone D., Romanelli B., Ierardi A. (2020). Postoperative Textiloma Mimicking Intracranial Rebleeding in a Patient with Spontaneous Hemorrhage: Case Report and Review of the Literature. Case Rep. Neurol..

[59] Akiboye F., Rayman G. (2017). Management of Hyperglycemia and Diabetes in Orthopedic Surgery. Curr. Diabetes Rep..

[60] Montemurro N., Perrini P., Mangini V., Galli M., Papini A. (2019). The Y-shaped trabecular bone structure in the odontoid process of the axis: A CT scan study in 54 healthy subjects and biomechanical considerations. J. Neurosurg. Spine.

[61] Corsalini M., Di Venere D., Sportelli P., Magazzino D., Ripa M., Cantatore F., Cagnetta C., De Rinaldis C., Montemurro N., De Giacomo A. (2018). Evaluation of prosthetic quality and masticatory efficiency in patients with total removable prosthesis: Study of 12 cases. Oral Implantol..

[62] Perrini P., Gambacciani C., Martini C., Montemurro N., Lepori P. (2015). Anterior cervical corpectomy for cervical spondylotic myelopathy: Reconstruction with expandable cylindrical cage versus iliac crest autograft: A retrospective study. Clin. Neurol. Neurosurg..

[63] Eriksson M., Kahari J., Vestman A., Hallmans M., Johansson M., Bergenheim A.T., Sandström M. (2019). Improved treatment of glioblastoma—changes in survival over two decades at a single regional Centre. Acta Oncol..

[64] Kyrgiou M., Kalliala I., Markozannes G., Gunter M.J., Paraskevaidis E., Gabra H., Martin-Hirsch P., Tsilidis K.K. (2017). Adiposity and cancer at major anatomical sites: Umbrella review of the literature. BMJ.

[65] Yang T.O., Cairns B.J., Kroll M.E., Reeves G.K., Green J., Beral V., Million Women Study Collaborators (2016). Body size in early life and risk of lymphoid malignancies and histological subtypes in adulthood. Int. J. Cancer.

[66] Petrelli F., Cortellini A., Indini A., Tomasello G., Ghidini M., Nigro O., Salati M., Dottorini L., Iaculli A., Varricchio A. (2020). Obesity paradox in patients with cancer: A systematic review and meta-analysis of 6,320,365 patients. BMJ.

[67] Lee J.Y., Lee S.W.H. (2018). Telemedicine Cost-Effectiveness for Diabetes Management: A Systematic Review. Diabetes Technol Ther..

[68] Montemurro N., Perrini P. (2020). Will COVID-19 change neurosurgical clinical practice?. Br. J. Neurosurg..

[69] Grassi F.R., Rapone B., Scarano Catanzaro F., Corsalini M., Kalemaj Z. (2017). Effectiveness of computer-assisted anesthetic delivery system (sta™) in dental implant surgery: A prospective study. Oral Implantol..

[70] Bao Z., Chen K., Krepel S., Tang P., Gong W., Zhang M., Liang W., Trivett A., Zhou M., Wang J.M. (2019). High Glucose Promotes Human Glioblastoma Cell Growth by Increasing the Expression and Function of Chemoattractant and Growth Factor Receptors. Transl. Oncol..

[71] Vasconcelos-Dos-Santos A., Loponte H.F., Mantuano N.R., Oliveira I.A., de Paula I.F., Teixeira L.K., de-Freitas-Junior J.C., Gondim K.C., Heise N., MohanaBorges R. (2017). Hyperglycemia exacerbates colon cancer malignancy through hexosamine biosynthetic pathway. Oncogene.

[72] Yu Y., Bao Z., Wang X., Gong W., Chen H., Guan H., Le Y., Su S., Chen K., Wang J.M. (2017). The G-protein-coupled chemoattractant receptor Fpr2 exacerbates high glucose-mediated proinflammatory responses of Muller glial cells. Front. Immunol..

[73] Marie S.K., Shinjo S.M. (2011). Metabolism and brain cancer. Clinics.

[74] Puzio-Kuter A.M. (2011). The Role of p53 in Metabolic Regulation. Genes Cancer..

[75] Yang C., Sudderth J., Dang T., Bachoo R.M., McDonald J.G., DeBerardinis R.J. (2009). Glioblastoma cells require glutamate dehydrogenase to survive impairments of glucose metabolism or Akt signaling. Cancer Res..

[76] Huang J., Hu J., Bian X., Chen K., Gong W., Dunlop N.M., Howard O.M.Z., Wang J.M. (2007). Transactivation of the epidermal growth factor receptor by formylpeptide receptor exacerbates the malignant behavior of human glioblastoma cells. Cancer Res..

[77] Zhou Y., Bian X., Le Y., Gong W., Hu J., Zhang X., Wang L., Iribarren P., Salcedo R., Howard O.M. (2005). Formylpeptide receptor FPR and the rapid growth of malignant human gliomas. J. Natl. Cancer Inst..

[78] Woolf E.C., Scheck A.C. (2015). The ketogenic diet for the treatment of malignant glioma. J. Lipid Res..

[79] Nathan D.M. (2007). Finding new treatments for diabetes—how many, how fast… how good?. N. Engl. J. Med..

[80] Zander T., Kraus J.A., Grommes C., Schlegel U., Feinstein D., Klockgether T., Landreth G., Koenigsknecht J., Heneka M.T. (2002). Induction of apoptosis in human and rat glioma by agonists of the nuclear receptor PPARgamma. J. Neurochem..

[81] He X., Esteva F.J., Ensor J., Hortobagyi G.N., Lee M.H., Yeung S.C. (2012). Metformin and thiazolidinediones are associated with improved breast cancer-specific survival of diabetic women with HER2+ breast cancer. Ann. Oncol..

[82] Bowker S.L., Majumdar S.R., Veugelers P., Johnson J.A. (2006). Increased cancer-related mortality for patients with type 2 diabetes who use sulfonylureas or insulin. Diabetes Care.

[83] Sesen J., Dahan P., Scotland S.J., Saland E., Dang V., Lemarié A., Tyler B.M., Brem H., Toulas C., Moyal E.C. (2015). Metformin inhibits growth of human glioblastoma cells and enhances therapeutic response. PLoS ONE.

[84] Xiao Z.X., Chen R.Q., Hu D.X., Xie X.Q., Yu S.B., Chen X.Q. (2017). Identification of repaglinide as a therapeutic drug for glioblastoma multiforme. Biochem. Biophys. Res. Commun..

[85] Perrini P., Montemurro N. (2016). Congenital absence of a cervical spine pedicle. Neurol. India.

[86] Montemurro N., Ortenzi V., Naccarato G.A., Perrini P. (2020). Angioleiomyoma of the knee: An uncommon cause of leg pain. A systematic review of the literature. Interdiscip. Neurosurg..

[87] Perrini P., Montemurro N., Iannelli A. (2013). The contribution of Carlo Giacomini (1840-1898): The limbus Giacomini and beyond. Neurosurgery.

[88] Pitter K.L., Tamagno I., Alikhanyan K., Hosni-Ahmed A., Pattwell S.S., Donnola S., Dai C., Ozawa T., Chang M., Chan T.A. (2016). Corticosteroids compromise survival in glioblastoma. Brain.

[89] Klement R.J., Champ C.E. (2014). Calories, carbohydrates, and cancer therapy with radiation: Exploiting the five R’s through dietary manipulation. Cancer Metastasis Rev..

[90] Panhans C.M., Gresham G., Amaral J.L., Hu J. (2020). Exploring the Feasibility and Effects of a Ketogenic Diet in Patients with CNS Malignancies: A Retrospective Case Series. Front. Neurosci..

[91] Poff A., Koutnik A.P., Egan K.M., Sahebjam S., D’Agostino D., Kumar N.B. (2019). Targeting the Warburg effect for cancer treatment: Ketogenic diets for management of glioma. Semin. Cancer Biol..

[92] Rapone B., Ferrara E., Montemurro N., Converti I., Loverro M., Loverro M.T., Gnoni A., Scacco S., Siculella L., Corsalini M. (2020). Oral Microbiome and Preterm Birth: Correlation or Coincidence? A Narrative Review. Open Access Maced. J. Med. Sci..

[93] Rieger J., Bahr O., Maurer G.D., Hattingen E., Franz K., Brucker D., Walenta S., Kämmerer U., Coy J.F., Weller M. (2014). ERGO: A pilot study of ketogenic diet in recurrent glioblastoma. Int. J. Oncol..

[94] Martin-McGill K.J., Marson A.G., Tudur Smith C., Young B., Mills S.J., Cherry M.G., Jenkinson M.D. (2020). Ketogenic diets as an adjuvant therapy for glioblastoma (KEATING): A randomized, mixed methods, feasibility study. J. Neurooncol..

